# The aspirin metabolite salicylate inhibits lysine acetyltransferases and MUC1 induced epithelial to mesenchymal transition

**DOI:** 10.1038/s41598-017-06149-4

**Published:** 2017-07-17

**Authors:** Harvey R. Fernandez, Sara K. Lindén

**Affiliations:** Department of Medical Biochemistry and Cell Biology, Sahlgrenska Academy, University of Gothenburg, Gothenburg, Sweden

## Abstract

MUC1 is a transmembrane mucin that can promote cancer progression, and its upregulation correlates with a worse prognosis in colon cancer. We examined the effects of overexpression of MUC1 in colon cancer cells, finding that it induced epithelial to mesenchymal transition (EMT), including enhanced migration and invasion, and increased Akt phosphorylation. When the clones were treated with the aspirin metabolite salicylate, Akt phosphorylation was decreased and EMT inhibited. As the salicylate motif is necessary for the activity of the lysine acetyltransferase (KAT) inhibitor anacardic acid, we hypothesized these effects were associated with the inhibition of KAT activity. This was supported by anacardic acid treatment producing the same effect on EMT. *In vitro* KAT assays confirmed that salicylate directly inhibited PCAF/Kat2b, Tip60/Kat5 and hMOF/Kat8, and this inhibition was likely involved in the reversal of EMT in the metastatic prostate cancer cell line PC-3. Salicylate treatment also inhibited EMT induced by cytokines, illustrating the general effect it had on this process. The inhibition of both EMT and KATs by salicylate presents a little explored activity that could explain some of the anti-cancer effects of aspirin.

## Introduction

MUC1 is a transmembrane mucin providing protective functions in epithelial cells against stressors including bacterial infection^1^ and chemical agents^2^. The large extracellular domain helps prevent bacterial binding to the epithelium, while the cytoplasmic subunit can provide signaling functions as well as translocating to the nucleus and regulating gene expression^3^. MUC1 levels vary in the gastrointestinal tract, being highly expressed in the stomach, but not in the colon, although expression increases during conditions of chronic inflammation such as ulcerative colitis^4^. These inflammatory conditions increase the risk of colon cancer^5^, and as *MUC1* acts as an oncogene in breast and pancreatic cancers^6, 7^, it might also promote carcinogenesis in the colon. Expression of human MUC1 in a mouse inflammation model was shown to increase the rate of progression to colon cancer^8^. Studies have found elevated levels of MUC1 in colon cancer are associated with greater invasiveness and poor prognosis^9, 10^, but it is undetermined whether this is causative.

Epithelial to mesenchymal transition (EMT), a mechanism whereby epithelial cells revert to a mesenchymal phenotype acquiring increased invasive/motile character, occurs during normal development and wound healing^11^. Cancer cells can undergo EMT, which may facilitate metastasis. MUC1 has been shown to be involved in EMT induction through a number of mechanisms, including interaction with β-catenin inducing upregulation of EMT inducing transcription factors such as Snail, Slug and Twist^12^. MUC1 also activates the Akt pathway^13^, which promotes EMT^14^. Indeed, MUC1 induces this process via Akt in non small cell lung cancer cells^15^.

An important negative regulator of Akt is the tumour suppressor phosphatase and tensin homolog (PTEN), which dephosphorylates phosphatidylinositol-3,4,5-trisphosphate (PtdIns(3,4,5)P), preventing the activation of Akt^16^. Mutations in PTEN lead to constitutive de-repression of the phosphoinositide 3-kinase (PI3K)/Akt pathway and increased proliferation and survival. The activity of PTEN is regulated by a number of post-translational modifications, including acetylation^16^. The lysine acetyltransferase (KAT) p300 and CBP Associated Factor (PCAF)/Kat2b acetylates PTEN in its C-terminal, reducing its ability to negatively regulate Akt. Therefore, inhibition of PCAF would be predicted to increase PTEN activity and reduce Akt signaling.

Aspirin (acetylsalicylic acid) is known to provide protection against colon cancer. Mechanisms proposed to explain this activity include inhibition of cyclooxygenases, induction of apoptosis, inhibition of NF-κB activity, upregulation of tumour suppressor genes and inhibition of mTOR signaling (reviewed in ref. 17). It has not been reported whether salicylate, the main metabolite of aspirin, inhibits KATs such as PCAF, however the relatively well characterized KAT inhibitor (KATi) anacardic acid (AA), 6-pentadyl-salicylic acid, contains the salicylate motif which is essential for its activity^18^. Anacardic acid inhibits PCAF, amongst other KATs, so we hypothesised that salicylate also exhibited this activity, albeit likely with lower potency. While micromolar concentrations of AA are required for KAT inhibition^18^, aspirin treatment can result in plasma salicylate concentrations in the low millimolar ranges^19^, potentially affecting KAT activity.

In this study we investigated the effects of overexpressing MUC1 in colon cancer cells with little endogenous expression of MUC1. We found that EMT was induced with MUC1 expression, and sodium salicylate treatment reversed this induction. This inhibition of EMT was likely caused by the reduction in Akt phosphorylation via the inhibition of PCAF. The results provide another explanation for the beneficial effects of aspirin against colon cancer.

## Results

### MUC1 overexpressing colon cancer cells underwent EMT

**Figure 1:**
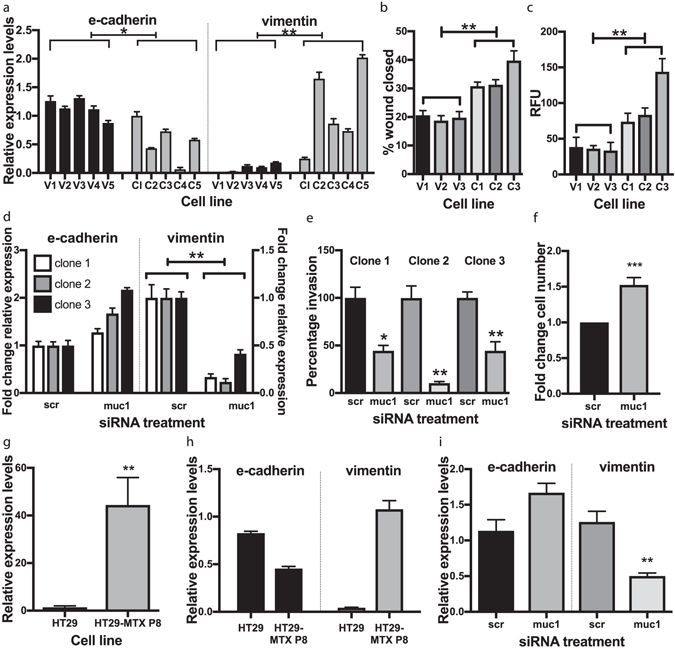
MUC1 induces EMT in the colon cancer cell line HT29: (**a**) EMT marker expression in MUC1 expressing clones. The relative mRNA expression levels of the epithelial (e-cadherin) or mesenchymal (vimentin) markers in MUC1 overexpressing clones (C1-C5) and vector control clones (V1-V5). Data are representative of three independent experiments with similar results. Clone 1 was arbitrarily chosen as the standard that all gene expression values were compared to. Values are mean ± SEM of (n = 3), and the Mann-Whitney U-test was performed to evaluate if these means differed between the MUC1 expressing clones and the control clones. *p < 0.05 and **p < 0.01. (**b**) MUC1 overexpressing clones exhibited increased migration and invasion. The migration rate of the MUC1 expressing clones relative to the vector control clones were analysed using an *in vitro* “wound healing” assay. A scratch was made in confluent cell layers, and the wounds photographed 72 h later. The percentage area of the wound closed was calculated. Data is representative of 3 independent experiments with similar results. Values are mean ± SEM (n = 3. **p < 0.01 by Student’s *t-*test. (**c**) The invasive ability of the MUC1 clones and vector control clones were evaluated using an *in vitro* gel-membrane scaffold simulating the extracellular matrix. Data are representative of 3 independent experiments with similar results. Values are mean ± SEM (n = 4). **p < 0.01 by Student’s *t-*test. RFU = relative fluorescence units. (**d**) MUC1 knockdown using siRNA reversed EMT. Clones overexpressing MUC1 were transfected with siRNA that specifically knocked down MUC1^1^ (muc1) and non-specific control siRNA (scr). Expression was measured 72 h after transfection of siRNA, and displayed as a fold change of the cells treated with MUC1 specific siRNA (muc1) compared to the non-specific control siRNA (scr). Data are representative of 3 independent experiments. **p < 0.01 by Student’s *t-*test. (**e**) The invasive ability of the siRNA treated cells were evaluated using an *in vitro* gel-membrane scaffold simulating the extracellular matrix. Values are mean +/− SEM (n = 3), expressed as a percentage compared to the scrambled control (scr) for each clone. *p < 0.05 and **p < 0.01 by Student’s *t-*test. (**f**) Fold change in cell numbers of the three clones combined with siRNA treatment ***p < 0.001 by Student’s *t-*test (**g**) MUC1 mRNA levels of the HT29 parental cell line that was used for transfection with the MUC1 construct, compared to the HT29-MTX-P8 subclone, which has higher endogenous expression of a number of mucins, including MUC1. Data are means of three individual experiments **p < 0.01 by Student’s *t-*test. (**h**) Comparison of the mRNA levels of EMT markers using real time PCR in the HT29 cells vs HT29-MTX-P8. The analysis was performed twice with similar results, data are means ± SEM (n = 3). (**i**) EMT marker expression after knockdown of MUC1 in the HT29-MTX-P8 cell line. Data points are the mean of three separate knockdown experiments ± SEM. **p < 0.01 by Student’s *t*-test.

To investigate the effects of overexpressing MUC1, the colon cancer cell line HT29 was transfected with a plasmid containing full length MUC1 with 23 tandem repeats, or empty vector control. MUC1 expression was confirmed via immunostaining, flow cytometry and PCR (Supplementary Fig. 1a–g). Five MUC1 overexpressing and five control clones were randomly chosen for initial experiments. The MUC1 expressing clones grew slower than controls (Supplementary Fig. 1h) and displayed morphological changes (Supplementary Fig. 2); they were elongated and less densely clustered than the controls: the average area of the individual cells of the three investigated MUC1 clones measured was 2.2 fold greater than those of the vectors (p < 0.05). The phenotype suggested EMT induction, and real time PCR demonstrated a decrease in mRNA levels of the epithelial marker e-cadherin, and an increase in the mesenchymal marker vimentin (Fig. 1a). As all MUC1 expressing clones displayed qualitatively similar marker changes, three clones were chosen for subsequent experiments, based on their morphological differences and magnitudes of EMT marker expression, with clone 1 showing the lowest EMT marker expression, clone 2 the highest and clone 3 intermediate.

Increased migratory and invasive capacity are characteristic of EMT. *In vitro* assays showed that the MUC1 expressing clones migrated faster than controls both after a wound healing scratch assay (p < 0.001, Fig. 1b) and by invasion through a gel mimicking the extracellular matrix (p < 0.05, Fig. 1c).

We used siRNA to knock down MUC1, to verify the EMT induction was a result of MUC1 expression. MUC1 knockdown caused the cells to increase growth rate (Fig. 1d) and change morphology (Supplementary Fig. 3), reversing the effects seen with MUC1 overexpression. Vimentin expression also decreased with MUC1 knockdown (p < 0.01, Fig. 1e), as did their invasive capacity (p < 0.05, Fig. 1f), verifying that EMT was induced by MUC1.

A subclone of the HT29 cell line, designated HT29 MTX-P8, has previously been isolated based on mucin expression^20^. We confirmed elevated MUC1 mRNA levels (Fig. 1g), and EMT was induced as indicated by vimentin and e-cadherin mRNA levels (Fig. 1h); these markers reversed expression when MUC1 was knocked down (Fig. 1i). This showed that increased endogenous expression of MUC1 resulted in the same effects as induced by overexpression in the MUC1 clones.

### MUC1 clones had higher Akt phosphorylation, and wortmannin reversed EMT marker expression, indicating the involvement of the Akt pathway

**Figure 2:**
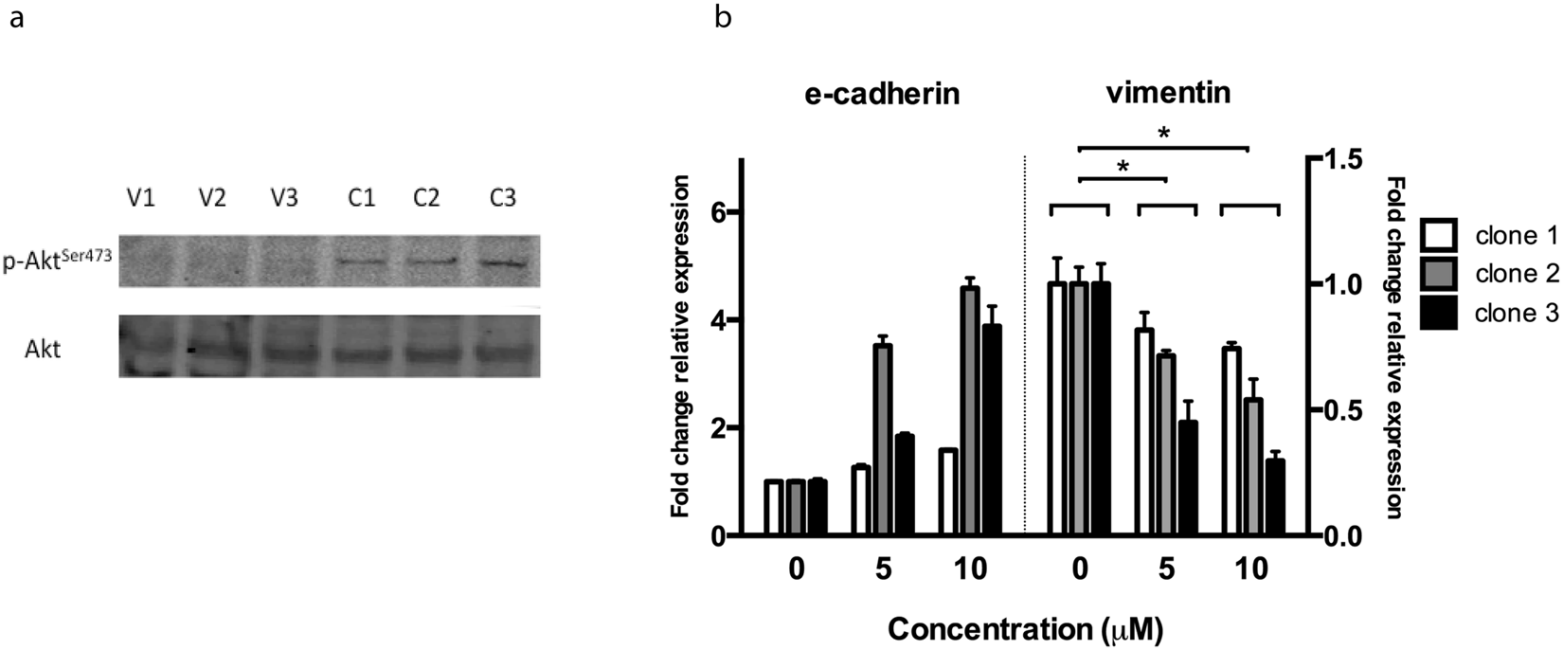
Akt phosphorylation is increased by MUC1 expression and EMT reversed by PI3K inhibition. (**a**) Western blot analysis of phosphorylated Akt and total Akt of the MUC1 expressing clones(C1-3) and vector control clones (V1-3). The membrane was sequentially probed with each primary and the corresponding secondary antibody, and scanned at their respective wavelengths. (**b**) Expression of EMT markers in MUC1 overexpressing clones with/without treatment with wortmannin for 12 h. Data are representative of three independent experiments, with means +/− SEM displayed (n = 3). *p < 0.05 by Student’s *t-*Test.

Since MUC1 has been shown to activate Akt^13^, a known promoter of EMT^14^, we examined the level of Akt phosphorylation by Western blot; the MUC1 clones did indeed display higher phosphorylation (Fig. 2a). The treatment of these clones with wortmannin, an inhibitor of PI3K, an upstream activator of Akt, resulted in a dose dependent increase and decrease in e-cadherin and vimentin mRNA levels respectively (Fig. 2b), confirming that the activation of Akt is involved in the induction of EMT in these cells.

### Sodium salicylate treatment reduced Akt phosphorylation and inhibited EMT

**Figure 3:**
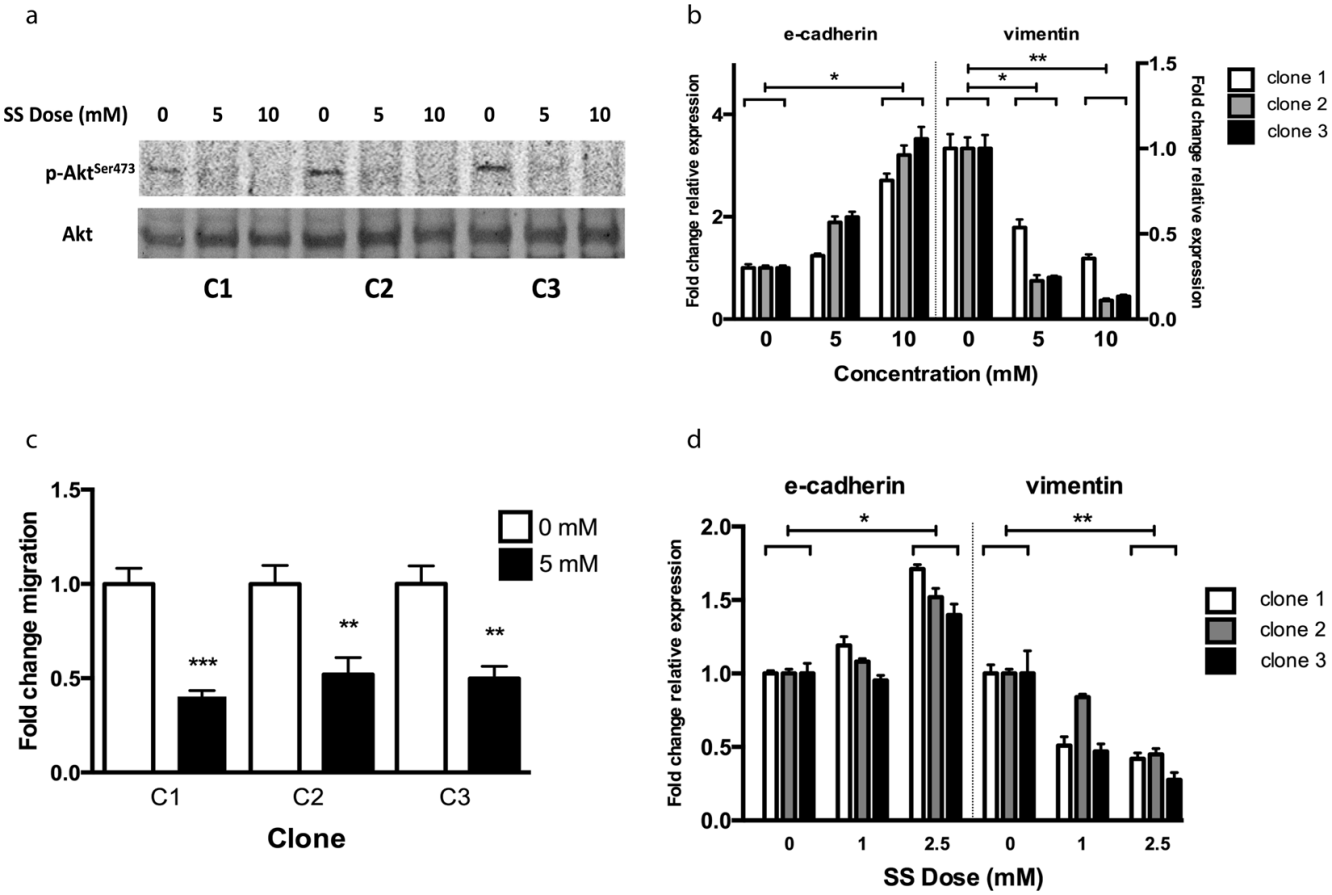
Sodium salicylate treatment reduces Akt phosphorylation and inhibits EMT. (**a**) The MUC1 expressing clones were treated with the indicated doses of SS for 10 h, and then harvested and analysed by Western blot. (**b**) The EMT marker mRNA expression after treatment of the MUC1 expressing clones with SS for 24 h. Data are representative of three separate experiments, presented here are means +/− SEM (n = 3). *p < 0.05 by one way ANOVA. (**c**) Migration of the MUC1 expressing clones with or without continued SS treatment measured 48 h after inducing the “wound” (n = 5). **p < 0.01, ***p < 0.001 by Student’s *t-*test. (**d**) EMT marker expression after low dose treatment for 4 days of the MUC1 expressing clones. A representative sample of 3 independent experiments shown, data displayed is the mean +/− SEM (n = 3). *p < 0.05 and **p < 0.01 by one way ANOVA.

The plasma half-life of aspirin is 20 minutes^21^, so many of its anti-cancer effects would likely be mediated by its main (more stable) metabolite, salicylate. Therefore, the MUC1 overexpressing HT29 clones were treated with sodium salicylate (SS), and this led to a reduction in Akt phosphorylation (Fig. 3a). Akt has been shown to phosphorylate vimentin and regulate its function^22^, and the SS-induced changes in cell morphology of the MUC1 expressing clones that were evident as early as 90 minutes after treatment with high doses (Supplementary Fig. 4) could reflect this. The cell junctions became less pronounced, and the cell clusters took on a more “branched” appearance. Injection of vimentin can cause changes in cell shape within 2 h^23^, so reduced phosphorylation by Akt could lead to these morphological changes, as vimentin is a major mesenchymal cytoskeletal protein.

Sodium salicylate treatment reversed EMT marker expression (Fig. 3b), and inhibited cell migration by 50% (p < 0.01, Fig. 3c). We also verified that this effect was not merely due to a reduction in expression of MUC1, as the SS treatment instead tended to increase MUC1 mRNA levels (Supplementary Fig. 5), which mirrors the slight increase in MUC1 protein levels seen in cells with aspirin treatment in a previous study from our laboratory^2^. To confirm that the results seen here were relevant to therapeutic doses, we treated the clones with concentrations equivalent to those that are achieved in plasma in patients treated with aspirin^19^, for a longer time period (4 days), and the mRNA levels of the EMT markers showed similar changes (Fig. 3d) to those seen with higher doses.

### Salicylate directly inhibits PCAF activity

**Figure 4:**
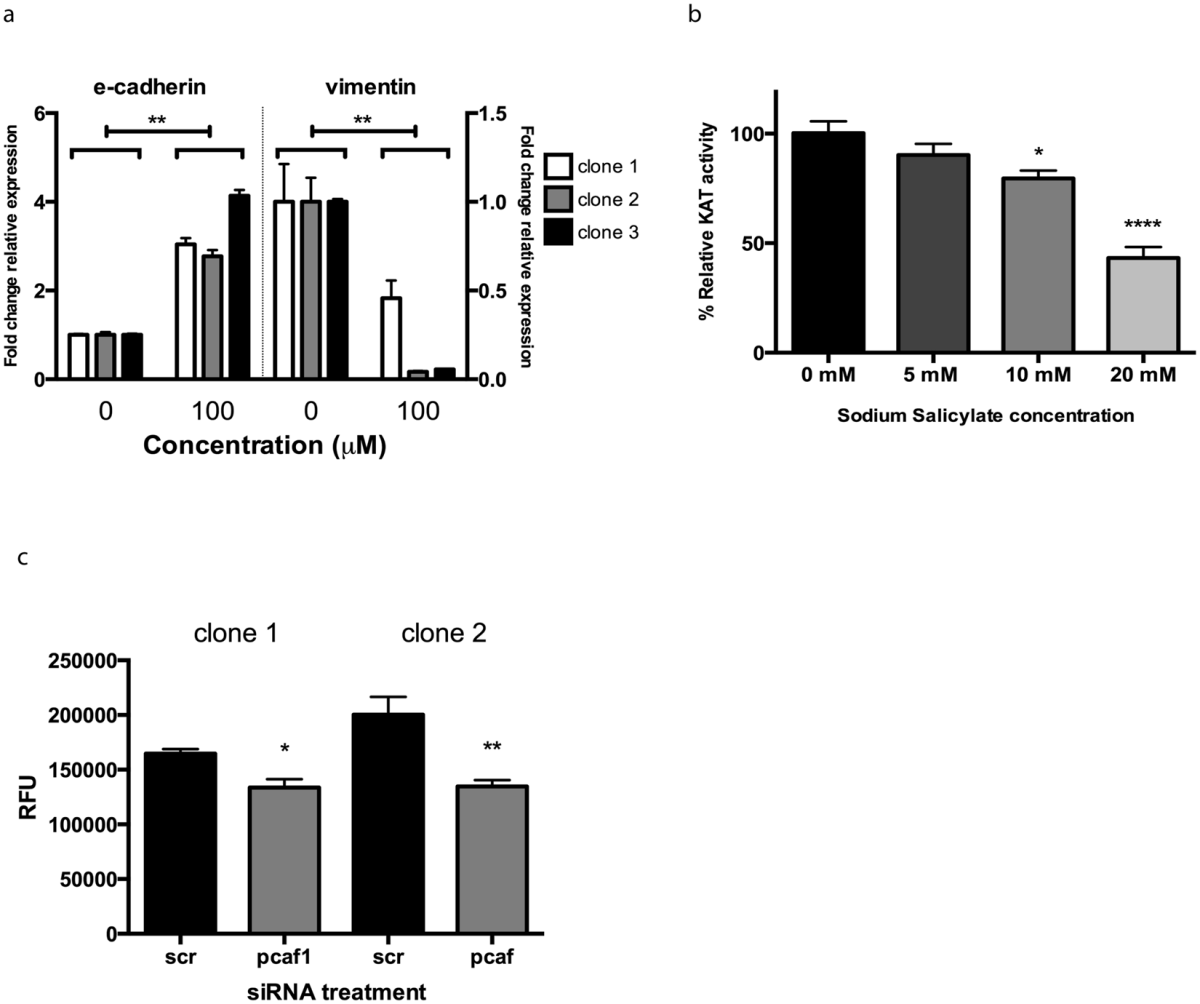
Anacardic acid also inhibits EMT, and SS directly inhibits PCAF activity. (**a**) EMT marker expression in the MUC1 expressing clones after anacardic acid treatment for 24 h **p < 0.01 by Student’s *t*-test. Data are representative of three independent experiments, presented are means +/− SEM (n = 3). (**b**) Recombinant purified PCAF was used in an *in vitro* KAT assay and acetyltransferase activity measured with and without addition of SS. Data are representative of three independent experiments, with the mean +/− SEM displayed (n = 4). *p < 0.05, ****p < 0.0001 by one-way ANOVA followed by Holm-Sidak’s multiple comparison test. (**c**) MUC1 expressing clones 1 and 2 were treated with either scrambled siRNA (scr) or siRNA targeting PCAF (pcaf), and used in the *in vitro* invasion assay(n = 5). *p < 0.05, *p < 0.01 by Student’s *t-*test. RFU = Relative fluorescence units.

Mutations in PI3K signaling components, including mTOR (which is activated by Akt^24^), are seen in many colorectal cancers^25^. Aspirin and salicylate have previously been shown to stimulate AMP activated protein kinase (AMPK)^26, 27^, which inhibits mTOR signaling. However, aspirin still affected mTOR even when AMPK was knocked down^26^, signifying the existence of an “AMPK independent” activity of aspirin; the reduction in Akt phosphorylation presented here describes such an activity. If SS was reducing Akt phosphorylation via PCAF inhibition, then anacardic acid (AA) would be predicted to produce the same effects. The MUC1 expressing clones were treated with 0.1 mM AA, and this did cause a reversal in expression of the EMT markers (p < 0.001, Fig. 4a). This supports the hypothesis that the effects of sodium salicylate on Akt phosphorylation and EMT are mediated via the inhibition of PCAF. To show that SS specifically targeted the activity of the PCAF enzyme, *in vitro KAT* assays were performed using commercially available purified active PCAF. These assays confirmed that SS directly inhibited PCAF activity (Fig. 4b). We then used siRNA to knock down PCAF, and these cells exhibited reduced invasion, verifying the requirement for PCAF activity to this process (Fig. 4c).

### The metastatic prostate cancer cell line, PC-3, showed reduced EMT marker expression and migration after sodium salicylate treatment, which likely involves Tip60 inhibition

**Figure 5:**
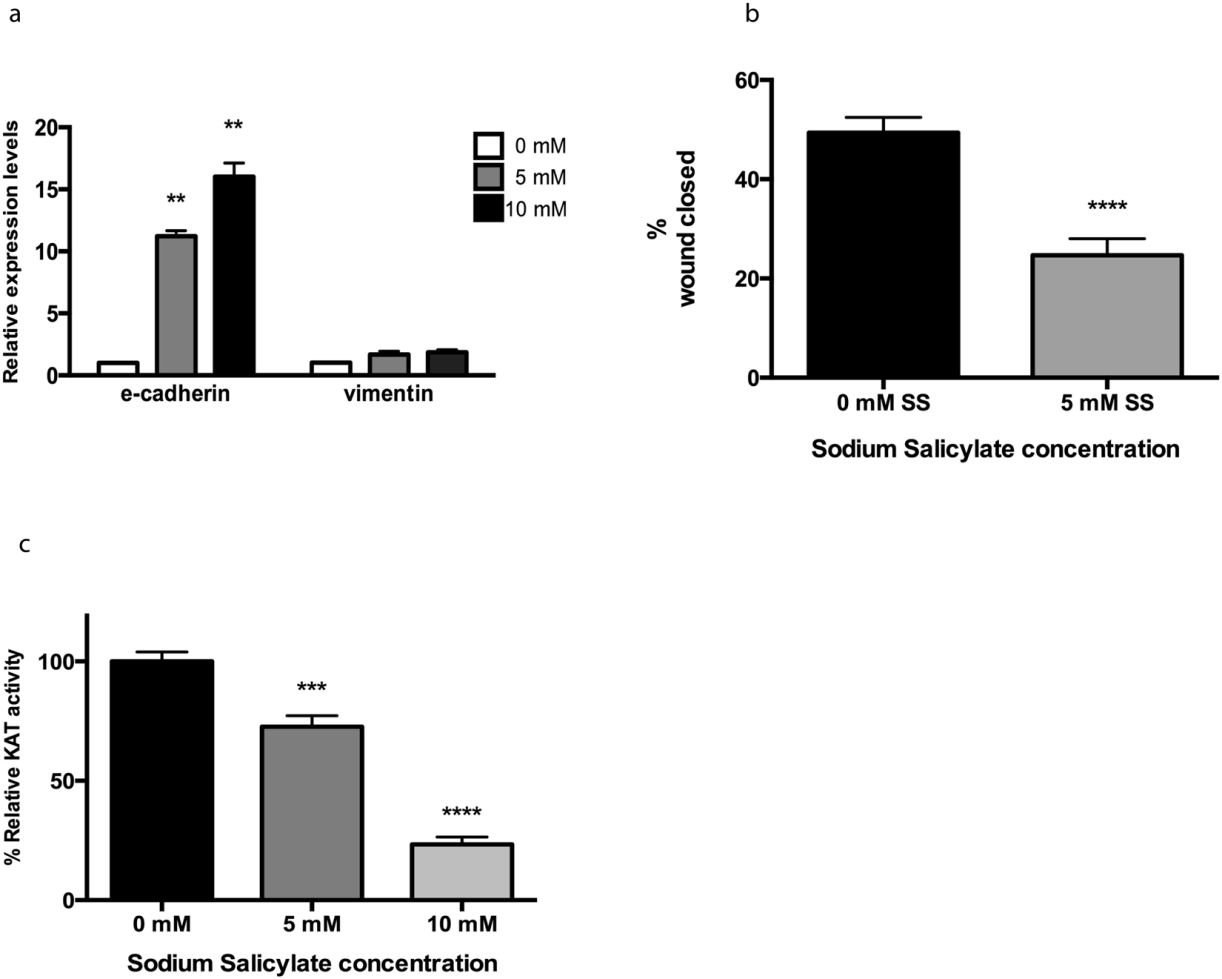
SS inhibits EMT in the PC3 prostate cancer cell line, and inhibits Tip60 activity. (**a**) EMT marker expression of the PC3 cell line treated with SS at the indicated doses for 24 h. Data presented are the means of three independent experiments, ± SEM. **p < 0.01 by Student’s *t-test*. (**b**) Migration of PC3 cells with or without treatment with SS ****p < 0.001 by Student’s *t-*test. (**c**) Acetyltransferase activity of the Tip60 lysine acetyltransferase was measured in the presence of the indicated doses of SS, using the *in vitro* KAT assay. Data are representative of three independent experiments, with the mean +/− SEM, displayed. ***p < 0.001 and ****p < 0.001 by one-way ANOVA followed by Holm-Sidak’s multiple comparison test.

These effects of SS and AA on EMT were examined in colon cancer cells. To investigate the generality of these effects, SS was used to treat a metastatic prostate cancer cell line, PC-3, which has previously been utilised as a model for EMT^28^. The mRNA levels of e-cadherin were 200x less than HT-29, while those of vimentin and n-cadherin were 1000x and 500x higher respectively (Supplementary Fig. 6), illustrating the mesenchymal character of these cells. In PC-3 cells e-cadherin mRNA increased 10 and 15 fold with 5 mM and 10 mM SS (p < 0.01 Fig. 5a). Reduced migration after treatment with 5 mM SS (which did not result in a significant reduction in cell numbers, see Supplementary Fig. 7) confirmed the reversal of EMT (p < 0.0001, Fig. 5b). As PC-3 cells are null for PTEN^29^, an alternative mechanism must be inhibiting EMT, which is supported by the qualitatively different response in EMT mRNA levels compared to the HT-29 cells: in HT-29, vimentin decreased, while e-cadherin increased, whereas in the PC-3 cells vimentin did not reduce expression while e-cadherin increased dramatically. Another KAT that is inhibited by anacardic acid is Tip60/Kat5^18^, which acetylates and activates the EMT inducing Twist^30^, a transcription factor required for EMT in PC-3 cells^31^. We therefore examined whether SS affected Tip60 activity using the *in vitro* KAT assay. Treatment with 10 mM SS reduced the activity of the enzyme by more than 75% (p < 0.001, Fig. 5c). Collectively, these data indicate that the inhibition of EMT in the PC-3 cell line is likely related to the reduced activity of Tip60, and demonstrates the broad nature of the effects of salicylate on EMT.

### Salicylate inhibits hMOF/KAT8 activity and reduces histone acetylation in cells

**Figure 6:**
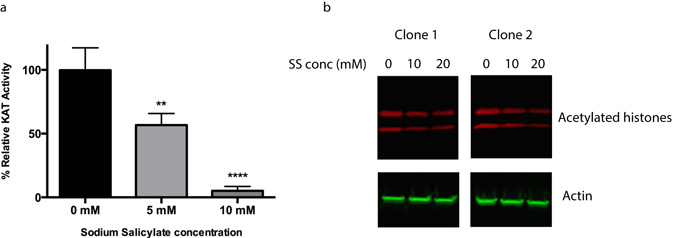
SS inhibits hMOF activity and reduces histone acetylation within cells. (**a**) The *in vitro* HAT assay was used to determine whether SS affected hMOF activity. The assay was performed three times with similar results, and a representative experiment shown. Data are means ± SEM (n = 4). **p < 0.01 and ****p < 0.0001 by one-way ANOVA followed by Holm-Sidak’s multiple comparison test. (**b**) Analysis of histone acetylation by Western blot (cropped image) after SS treatment of the MUC1 expressing clones for 14 h. The membrane was sequentially stained with the primary and their respective secondary antibodies and scanned at their respective wavelengths. Histones are displayed in the top panel; the top band is histone H3, and the bottom band histone H4. Densitometric analysis of the histone intensity normalised to actin are displayed in Supplementary Fig. 10a. The complete blots are displayed in Supplementary Fig. 10b.

Another KAT related to Tip60 is hMOF/KAT8, the reduced expression of which has been observed in colorectal and gastric cancer^32^. One characteristic of cancers with lower KAT expression might be an increased sensitivity to drugs targeting hMOF activity. This would have profound effects as hMOF is the KAT responsible for the majority of histone H4K16 acetylation, a modification with essential roles in chromatin function^33^. Indeed, using the KAT assay, SS inhibited hMOF (p < 0.001, Fig. 6a). To determine whether acetylation within cells was affected by SS treatment, MUC1 clones were treated with SS, and they displayed reduced histone acetylation (Fig. 6b), replicating in a cellular context the inhibitory activity of SS from the *in vitro* assays.

### Sodium salicylate reverses EMT induced by cytokine treatment

EMT can be induced in cancer cell lines by treatment with cytokines, so we investigated whether SS would inhibit such mechanisms of EMT induction. HT-29 cells were treated with either (1) TGFβ1, (2) EGF/TGFβ1, which induces EMT via Akt in intestinal epithelial cells^34^ or (3) EGF/basic fibroblast growth factor (bFGF). Cells were treated for at least 2 weeks, and then SS was added for 48 h. The cytokine treatment resulted in morphological changes similar to the overexpression of MUC1 (compare with Supplementary Fig. 1a); SS treatment reversed these changes and caused the cells to grow in more dense clusters (Supplementary Fig. 8a). Analysis of mRNA expression of the EMT markers confirmed that SS increased mRNA levels of e-cadherin and decreased vimentin (Supplementary Fig. 8b). This effect was also observed when growing the cells continuously in the presence of a lower dose (1 mM) of SS (Supplementary Fig. 8c). The results demonstrate that SS also inhibits EMT induced via mechanisms other than MUC1 expression.

## Discussion

We show here that MUC1 induces EMT in colon cancer cells, which likely involved Akt activation, and this was inhibited by treatment with salicylate. The results indicate that this effect on EMT was associated with the inhibition of lysine acetyltransferase activity and the consequent reduction on Akt phosphorylation. Furthermore, salicylate reversed EMT in prostate cancer cells as well as those treated with cytokines, illustrating the broad nature of this effect.

MUC1 has been defined as an oncoprotein resulting from its ability to promote cancer progression^6, 7^. The results here suggest a possible mechanism, the induction of EMT, behind the previously reported associations between MUC1 expression and poor colon cancer prognosis. While MUC1 induces EMT in other types of cancers, this is the first demonstration in colon cancer cells, of which the effects of ectopic MUC1 expression have not been extensively examined.

Initially, epidemiological studies uncovered an association between aspirin consumption and reduced risk of developing cancer, and subsequently aspirin was found to increase the survival of patients already diagnosed with colorectal cancer^35^. While the inhibition of EMT could be considered more relevant to the latter situation, it is still pertinent to the former, as it has been shown that there is expression of vimentin and loss of e-cadherin in benign adenomas, the precursors to adenocarcinoma^36^. While low doses of aspirin taken for cardiovascular disease prevention (<100 mg) likely doesn’t acutely affect KAT activity, it is possible that the cumulative effect of even slight alteration of expression of EMT associated proteins over a number of years could have an effect on the progression to cancer. Furthermore, expression of the EMT-inducing transcription factors Snail and Twist, and a corresponding reduction in e-cadherin were detected in adenomas^37^, and Akt expression was demonstrated to be an early event in sporadic colon carcinogenesis in both human samples and rodent models^38^. Another salicylic acid, 5-aminosalicylic acid, was found to significantly increase membranous e-cadherin staining in adenomas of patients^39^. This is consistent with our observations that SS increases e-cadherin mRNA levels in both colon cancer and prostate cancer lines, and is particularly relevant because the loss of e-cadherin alone is sufficient for the induction of EMT^11^.

As a change in histone acetylation would result in changes in gene expression, we checked that the inhibition of EMT was not merely due to a decrease in MUC1 expression. Indeed, mRNA levels of MUC1 slightly increased, in line with results from a previous study from our laboratory that showed that aspirin treatment of cells led to a slight increase in the MUC1 protein^2^. The inhibition of acetylation being responsible for the effect on EMT was also confirmed by anacardic acid treatment producing the same results.

When mice were treated with aspirin that was approximately equivalent to a low dose in humans, plasma salicylate concentrations peaked at 1 mM^40^. In humans, high dose treatments used for analgesic purposes attain concentrations^17^ in excess to what we found inhibited EMT. While the doses used in the *in vitro* KAT assays were much higher, the clear dose dependence of the inhibition demonstrated that it was specific towards the enzyme. A paper published just before the submission of this manuscript that showed salicylate inhibits the KATs CBP/p300^41^, also found salicylate-coA, a major intermediate formed during salicylate metabolism, exhibited an almost 30 fold increase in KAT inhibitory activity. So while our *in vitro* KAT assays showed that higher concentrations of salicylate were required for inhibition compared to the concentration required to inhibit EMT, the formation of this intracellular intermediate provides an explanation for this disparity. The most pertinent benefit of the KAT inhibitory activity of aspirin/salicylate on cancer would be as a therapeutic, and this is supported by studies employing higher doses than those used for cardiovascular disease control. For example, treatment with doses > 300 mg have been shown to have positive effects on mortality in trials within as little as 3 years^42^; another large randomized trial found treatment with the same dose was necessary for anti-cancer effects^43^ while treatment of carriers of hereditary colorectal cancer with 600 mg/day substantially reduced cancer incidence^44^.

Two important studies analysed the types of colon cancers that responded to aspirin treatment, and discovered that only a subset of tumours were sensitive: those containing activating mutations of *PI3KCA* ^45, 46^, which results in a constitutively upregulated PI3K/Akt/mTOR pathway. Elucidating the mechanism behind these observations has up to this point remained elusive, but the results presented here, showing that SS reduces Akt phosphorylation provides a logical explanation. Furthermore, the inhibition of KATs by SS introduces different treatment possibilities, as they have many important functions that can be utilised. Tip60 and hMOF are involved in DNA repair^47, 48^, and the downregulation of hMOF^32^ and Tip60^49^ are associated with the progression of colorectal cancer. Consequently, conventional therapeutics using DNA damaging agents could benefit from co-treatment with salicylate, as this would selectively target these tumours more effectively compared to surrounding tissue with normal levels of these KATs. Related to this concept, it has been shown that anacardic acid treatment made cells more sensitive to ionising radiation^50^.

The prevalence of histone deacetylase inhibitors as cancer therapeutics illustrate the importance of acetylation, with KATs playing important roles in cancer biology. A close interaction partner of the Tip60 complex was found to promote colorectal cancer development^51^. Furthermore, β-catenin, activated after Adenomatous polyposis coli (APC) mutation which occurs in the majority of colon cancers, interacts with and requires Tip60 for activity^52^. Another study showed that PCAF acetylates, stabilizes and activates β-catenin^53^. The inhibition of the activities of these two KATs affecting an integral initiating pathway of colon cancer helps clarify the profound beneficial effects of aspirin.

Aspirin has long been known to inhibit NF-κB activity^54^, and this transcription factor plays many roles in the induction of EMT^55^. In our study, the EMT marker expression reversion could be a direct consequence of NF-κB inhibition, as vimentin has been shown to be one of its transcription targets, while it simultaneously suppresses e-cadherin^56^. Anacardic acid has also been shown to inhibit NF-κB activity, by inhibiting the phosphorylation and degradation of its negative regulator IκB^57^. This is consistent with our results, which indicate that the reduction in transcription of vimentin is related to the inhibition of Akt activity, as Akt can phosphorylate IκB and activate NF-κB^58^. One study did find that NF-κB was activated, not inhibited, by aspirin in HT-29 cells and xenografts^40^. Those cells were, however, the equivalent of our untransfected parental cell line, which did not have the increased Akt phosphorylation that would stabilise NF-κB, and explains the difference between their observations and our model.

Akt and AMPK have antagonistic effects on mTOR signalling^59^. Both aspirin^26^ and salicylate^27^ activate AMPK, which inhibits mTOR signaling. Our finding that salicylate also inhibits Akt phosphorylation would similarly affect this pathway; furthermore, Akt downregulates AMPK activity^60^, indicating that the effects of salicylate on AMPK could also, at least partly, relate to the inhibition of Akt. The advantage that SS has over AA and its derivates is that its pharmacology is well studied; anacardic acid, in contrast, has only been investigated *in vitro* and in animal models. While SS is a much less potent KAT inhibitor, millimolar plasma concentrations are achievable. Other mechanisms in addition to the KAT inhibition could also be involved in the effect on EMT. Aspirin has been shown to downregulate the SP family of transcription factors^61^, and SP1 is involved in the induction of EMT^62^. Moreover, the enzyme activities of matrix metalloproteinases 2 and 9, which are integral to EMT progression, are directly inhibited by anacardic acid and salicylic acid^63^. As salicylate was also recently shown to inhibit the KATs CBP/p300, this could also have a role in inhibiting EMT, as they are also associated with its induction^64, 65^. KATs are also involved in numerous cancer promoting functions in addition to EMT^66^, so their inhibition would target oncogenesis from multiple angles. Therefore, the inhibition of KAT activity by salicylate could further contribute to the anti-cancer effects of aspirin in conjunction with the pleiotropic effects on multiple biochemical pathways already reported^17^.

This study has yielded findings that help clarify many previous observations concerning colon cancer progression, prevention and treatment. Firstly, the expression of MUC1 in colon cancer cells induces EMT, which indicates a causative role behind MUC1 expression and its relationship to the severity of colon cancer. Secondly, salicylate, the main breakdown product of aspirin, displays KAT inhibitory activity in concentrations similar to those achieved in patients. This has wide implications considering the central role in many biological pathways that KATs play. The effect on the Akt pathway and the ensuing reversal of EMT explain many previous observations regarding the effects of aspirin on colon cancer. The results uncover new mechanisms behind the anti-cancer effect of aspirin, and this knowledge could allow novel strategies and combined therapies to be deployed.

## Materials and Methods

### Cell culture

The colorectal cancer cell line HT29, containing very low levels of MUC1 protein, was used to overexpress MUC1, and was maintained in RPMI 1640 medium with penicillin/streptomycin and 10% fetal bovine serum (Lonza, Basel, Switzerland). A subclone of HT29, designated “HT29 MTX P8”^20^ and the prostate cancer cell line PC-3 were similarly cultured. The plasmid, pdEST47, containing full length human MUC1 with 23 tandem repeats was transfected into HT29 using Lipofectamine® 2000 (Life Technologies, Carlsbad, CA, U.S.A); the vector without an insert was transfected to obtain control clones. Stable clones were isolated from single colonies under geneticin (Life Technologies) selection.

MUC1 protein overexpression was confirmed by immunostaining. Briefly, clones were grown in chamber slides, washed in PBS, fixed in 4% formaldehyde in PBS for 20 min on ice, then blocked for 30 min with blocking buffer (PBS + 0.05% Tween and 10% FBS). The cells were probed with a 1/200 dilution of the BC2 antibody^1^ for 1 h at room temperature, washed three times with blocking buffer, probed with AF594 secondary antibody at 1/1000 for 1 h and washed three times in blocking buffer before being mounted.

### Drug treatments (all drugs from Sigma, St. Louis, MO, U.S.A.)

Sodium salicylate (SS) was dissolved in deionized water and then filter sterilized; anacardic acid (AA) and wortmannin were dissolved in DMSO before addition to cells. Control cells were treated with vehicle.

### Cytokine treatments to induce EMT

Cells were grown in 5% FBS and treated with either (a) Transforming growth factor beta-1 (TGFβ1) (Sigma) (10 ng/ml); (b) Epidermal growth factor (EGF) (Sigma) (50 ng/ml) plus TGFβ1 (10 ng/ml), or (c) EGF (Sigma) (20 ng/ml) plus basic fibroblast growth factor bFGF (Sigma) (10 ng/ml). Cells were grown continuously and passaged in the presence of these cytokines for at least 2 weeks before addition of SS. For long term SS treatment, the cells were passaged continuously with 1 mM SS for at least 2 weeks before harvesting for EMT marker mRNA analysis.

### cDNA synthesis, Real Time PCR

Total RNA was isolated using Trizol® (Life Technologies), and 5 µg of total RNA was treated with DNase I (Life Technologies), in the presence of SUPERase^.^In^TM^ (Life Technologies) for 30 minutes at 37 °C; EDTA was then added to 5 mM and the DNase I heat inactivated at 75 °C for 10 minutes. After adding MgCl_2_ to 5 mM, cDNA was generated using Superscript III (Life Technologies) and oligo dT following the manufacturer’s instructions. Real time PCR was carried out using SSOfast^TM^ EvaGreen® Supermix (Bio-Rad, Hercules, CA, U.S.A.), and relative expression levels of EMT marker genes *CDH1* (encoding E-cadherin), *CDH2* (encoding n-cadherin) and *vim* (encoding vimentin) determined with the Bio-Rad CFX Manager^TM^ software, using the reference genes glyceraldehyde-3-phosphate dehydrogenase (GAPDH), glucose-6-phosphate isomerase (G6PI) and small nuclear ribonucleoprotein D3 (SNRPD3). Primers were designed using the Primer 3 program^67^. Primer efficiencies were calculated and input into the software. Primer sequences and efficiencies are listed in Table S1. The gene expression was normalised to the two housekeeping genes, and the gene expression changes calculated using the ∆∆CT method as the efficiencies of the primers were all similar. The gene expression changes were displayed as a fold change compared to the control set at a value of 1 unless otherwise stated.

### RNAi knockdown experiments

Cells were seeded into 6 well plates at 5×10^5^ cells/well. After 24 hours, 60 pmoles of 2′-hydroxyl DsiRNA against the target sequence NNGCACCGACUACUACCAAGA or siCONTROL (SC) non-targeted siRNA#2 (Dharmacon) in 7.5 µl of Lipofectamine® RNAiMAX Reagent (Life Technologies) were transfected following the manufacturer’s instructions. Both the MUC1 targeting sequence and the scrambled control have previously been verified to be specific and not give off target effects^1^.

### Western Blotting

Cell lysates were prepared by harvesting in lysis buffer (2% SDS, 20% glycerol, 50 mM Tris-HCl, 150 mM DTT, pH 7.4), separated on 4–20% SDS PAGE gels (Bio-Rad) and transferred to nitrocellulose membranes by electroblotting, probed with the primary antibodies: mouse anti-phospho-Akt (Ser473), clone 6F5 (Millipore), rabbit anti-acetyl lysine (Millipore), rabbit anti-Akt1/2/3 (Santa Cruz Biotechnology, Dallas, TX, U.S.A.), mouse anti-β-actin (abcam, Cambridge, U.K.) followed by secondary antibodies conjugated with infrared probes, and scanned on an Odyssey CLx Infrared Imaging System (Li-COR Biosciences, Lincoln, NE, U.S.A.). For histone acetylation Westerns, the cells were treated with 10 mM sodium butyrate to increase the background levels of acetylation, co-treated with SS at the listed concentrations, and harvested after 8 h.

### Cell area calculations

Eight separate areas of cells were traced using the freehand drawing tool in ImageJ^68^, with a minimum of 250 cells per MUC1 clone or vector control, and the area calculated per cell for each.

### Migration assays

Cells were seeded in 96 well plates, at 40,000 cells/well. After 24 hours, media was removed and replaced with serum free media, and left for another 18 h. A scratch was made with a pipette tip, and the cells in each well photographed on an EVOS® XL Core Cell Imaging System (Life Technologies). After 48–72 h each well was photographed again, and the migration area calculated by tracing using the freehand drawing tool in ImageJ, and determining the percentage of the wound closed after normalising the initial wound sizes for all cell lines/treatments. For the salicylate treatment, cells were seeded, then after 24 h the media was replaced with serum free media with or without drug, the scratch made 12 hours later, and monitored as above.

### Invasion assay

The QCM^TM^ 96-Well Collagen-Based Cell Invasion Assay (Merck-Millipore, Billerica, MA, USA) was used following the manufacturer’s instructions: sixty thousand cells suspended in serum free RPMI containing 5% BSA were loaded in the upper well, and media with serum (10%, diluted in RPMI) in the bottom well. The cells were incubated for 12–14 h before analysis following the manufacturer’s instructions.

### KAT assays

A fluorescent KAT assay from Active Motif (Carlsbad, CA, U.S.A.) was used to assay the activity of purified recombinant PCAF (KAT2B), Tip60 (KAT5) (both from (Caymen Chemicals, Ann Arbor, MI, U.S.A.) or KAT8/hMOF (Sigma) with or without sodium salicylate addition, following the manufacturer’s instructions. Enzyme concentrations were 2.5 nM PCAF, 10 nM Tip60 or 10 nM hMOF, and histone peptide H3 was used as the substrate for PCAF, or H4 for Tip60 and hMOF. Both PCAF and Tip60 were directly used in the assay, however the KAT8/hMOF enzyme preparation contained component/s that interfered with the assay, so was buffer exchanged with PBS at least 500x using an Amicon Ultra 10 kDa cutoff centrifugal filter (Merck-Millipore) before a final 2x dilution with assay buffer. Both PCAF and Tip60 did not display high levels of autoacetylation, so the blank controls were co-incubated without histones as in the kit instructions, however hMOF did have relatively high levels of acetyltransferase activity contributed by autoacetylation, so the blank wells were prepared by adding stop solution to the blank wells (those without and with SS) at time 0, and the acetyltransferase activity measured was the cumulative signal of both autoacetylation and histone acetylation.

### Statistics

Data are expressed as Mean ± SEM in graphs and statistical tests were performed using GraphPad Prizm 5.0 (GraphPad Software Inc.) and IBM SSPS 22 (IBM corp.). The Mann-Whitney U-test or Student’s T-test (depending on data distribution) was used to make comparisons between clones with and without MUC1 overexpression as well as between treatment groups. Data for real time EMT markers required log transformation to achieve a data distribution suitable to perform the statistical test, however the graphs were presented as a fold change in expression for clarity. For multiple comparisons, one way ANOVA was performed followed by Holm Sidak’s multiple comparison test.

## Electronic supplementary material

Supplementary Information
