# Supplementary material for: The aspirin metabolite salicylate inhibits lysine acetyltransferases and MUC1 induced epithelial to mesenchymal transition
Source: Sci Rep. 2017 Jul 17;7:5626. doi: 10.1038/s41598-017-06149-4 (PMC5514058; doi:10.1038/s41598-017-06149-4)
Supplement: Supplementary file 1 — Supplementary Information [file 41598_2017_6149_MOESM1_ESM.pdf]

The aspirin metabolite salicylate inhibits lysine acetyltransferases and MUC1 induced epithelial to mesenchymal transition.

Harvey R. Fernandez and Sara K. Lindén.

Supplementary Information

Table S1: Primer information

Gene name	Sequence	Efficiency
Glyceraldehyde-3 phosphate dehydrogenase (GAPDH) **Fwd**	CCCTCCGGGAAACTGTGGCG	103.6%
Glyceraldehyde-3 phosphate dehydrogenase (GAPDH) **Rev**	GCAGTGGGGACACGGAAGGC	
Small nuclear ribonucleoprotein D3 (SNRPD3) **Fwd**	GAGGACAACATGAACTGCCA	103.4%
Small nuclear ribonucleoprotein D3 (SNRPD3) **Rev**	TAACATGGGTGCGTTCTTCA	
CDH1 (E-cadherin) **Fwd**	TGGCTGAGCTGAACACATTTGCCC	100.4%
CDH1 (E-cadherin) **Rev**	CCCCTACCCCTCAACTAACCCCC	
CDH2 (N-cadherin) **Fwd**	CACTGCTCAGGACCCAGAT	106.4%
CDH2 (N-cadherin) **Rev**	TAAGCCGAGTGATGGTCC	
Vimentin **Fwd**	GACAGGATGTTGACAATGCG	101.2%

Vimentin **Rev**	GTTCCTGAATCTGAGCCTGC	
Muc1 primers	Propriety (Qiagen)	

Supplementary Figure Legends

Supplementary Figure 1: MUC1 clones. a-d: The cells were stained with the BC2 antibody[1]. The localisation of the signal around the cell membrane indicates the functionality of the MUC1 protein expressed from the transfected construct: a – parental HT-29 cell line, **b-d** – clones 1-3. **e,f**: Flow cytometry analysis of clones 1 and 2 stained with the same BC2 antibody (green curves, vs HT-29, black curves). **g**: mRNA levels of MUC1 in parental cell line vs 3 clones. **h**: growth curves of MUC1 expressing clones 1-3 vs vector clones 1-3.

Supplementary Figure 2: Cell morphology of the MUC1 clones were altered. Morphology of three of the HT29 vector control clones (a-c) and three MUC1 expressing clones (d-f)

Supplementary Figure 3: Knockdown of MUC1 reversed effects of its expression. Morphology of the MUC1 expressing clones after siRNA treatment with a non specific sequence (scr) or a MUC1 targeting sequence (muc1), 72 h after transfection. Scale bar: 100μM

Supplementary Figure 4: **Sodium salicylate induced morphological changes.** Morphology of two of the MUC1 expressing clones after 90 minutes treatment with 10 mM or 20 mM sodium salicylate. Even after such a short treatment time, morphological changes are evident, with the cell clusters taking a more "branched" character, with intercellular junctions less clearly visible.

Supplementary Figure 5: **SS treatment did not reduce MUC1 mRNA.** Measurement of mRNA levels of MUC1 after 24 h treatment with 5 mM sodium

salicylate confirmed that the inhibition of EMT was not simply due to suppression of MUC1 expression. Results presented are representative of 3 independent experiments, data are means ± SEM (n=3).

Supplementary Figure 6: **EMT marker expression in PC3 vs HT-29 cells.** mRNA expression levels of epithelial (e-cadherin) and mesenchymal markers (n-cadherin and vimentin) in the HT29 colon cancer cell line versus the prostate cancer cell line PC3. Results are representative of 3 independent experiments, with means ± SEM (n=3).

Supplementary Figure 7: Cell viability of PC3 cells after SS treatment. Confluent PC3 cells were treated with 5 mM SS for 48 hours in serum free media, to mimic the migration assay conditions. The cells were then trypsinised and counted using trypan blue exclusion, and data presented as a fold change in cell numbers compared to the untreated cells. The SS treatment caused a slight, not statistically significant, reduction in cell numbers. Data points are the means ± SEM of three wells.

Supplementary Figure 8: EMT induced by cytokine treatment is inhibited by SS. (a) HT29 cells were were plated at equal densities and treated with either: 1) endothelial growth factor (EGF) and basic fibroblast growth factor (bFGF); (2) EGF/transforming growth factor beta 1 (TGFβ1) or (3) TGFβ1 alone for at least two weeks, and then co-treated with 4 mM sodium salicylate for 48 h. Cytokine treatment alone resulted in a morphology similar to that displayed by MUC1 clones 1 and 3. The treatment with SS, however, caused the cells to grow in more tightly packed clusters, similar to untreated HT29 cells. (b) EMT marker expression induced by cytokine treatment was reversed by the treatment of SS (4 mM) for 48 h. The control is the untreated HT29 cell lines showing the levels of expression the EMT markers before cytokine treatment. The experiment was performed three times with similar results, and a representative dataset shown. Data are mean +/-

SEM (n=3). (c) Analysis of EMT markers after continuous treatment with cytokines with or without 1mM SS. The cells were grown in these conditions for at least 3 weeks, and harvested on three separate occasions with consistent results, with one representative experiment shown. Results are means ± SEM (n=3).

Supplementary Figure 9: (a): Densitometry analysis of the intensity of the histone bands, normalized to actin, in Figure 6b. (b) Complete blots from Figure 6b, of acetylated histones after treatment with SS. The panels in Figure 6b are indicated by the white boxes.

Supplementary Figure 1

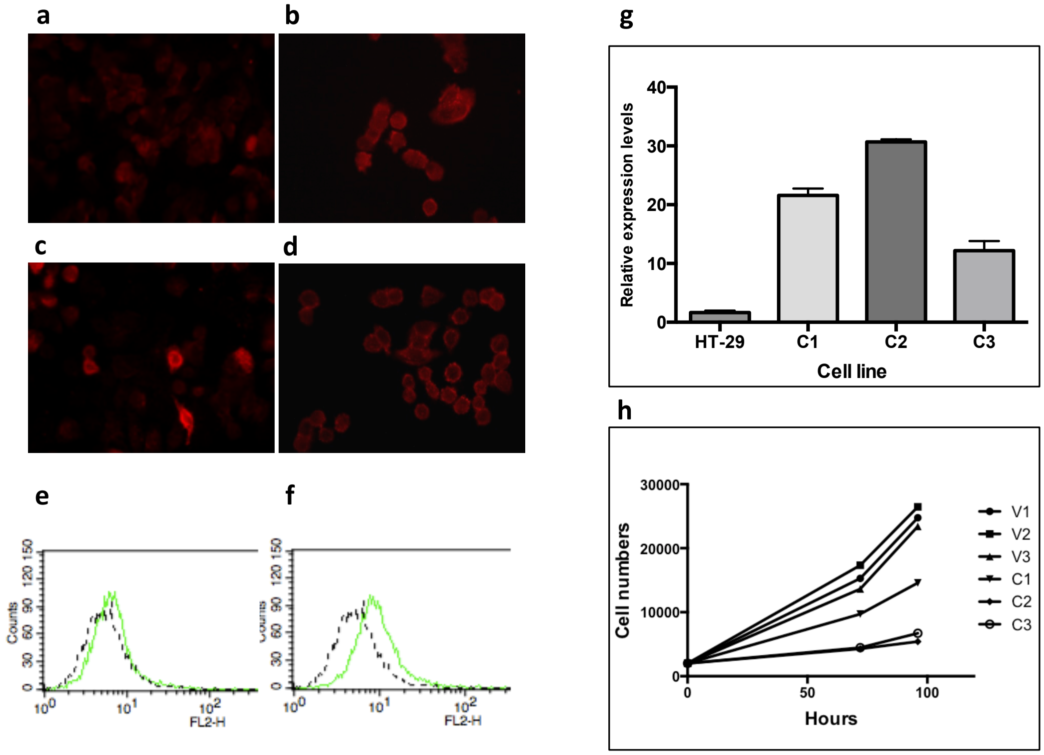

Supplementary Figure 2

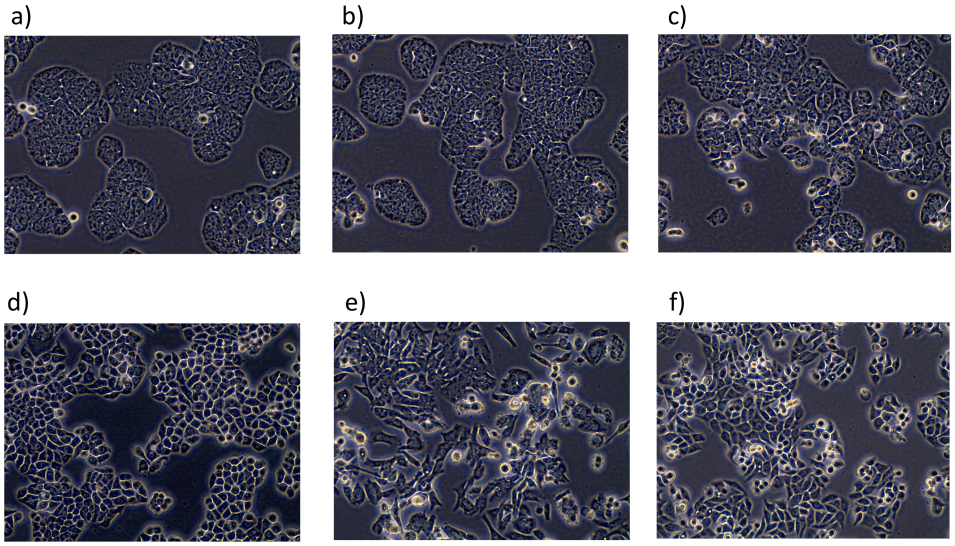

Supplementary Figure 3

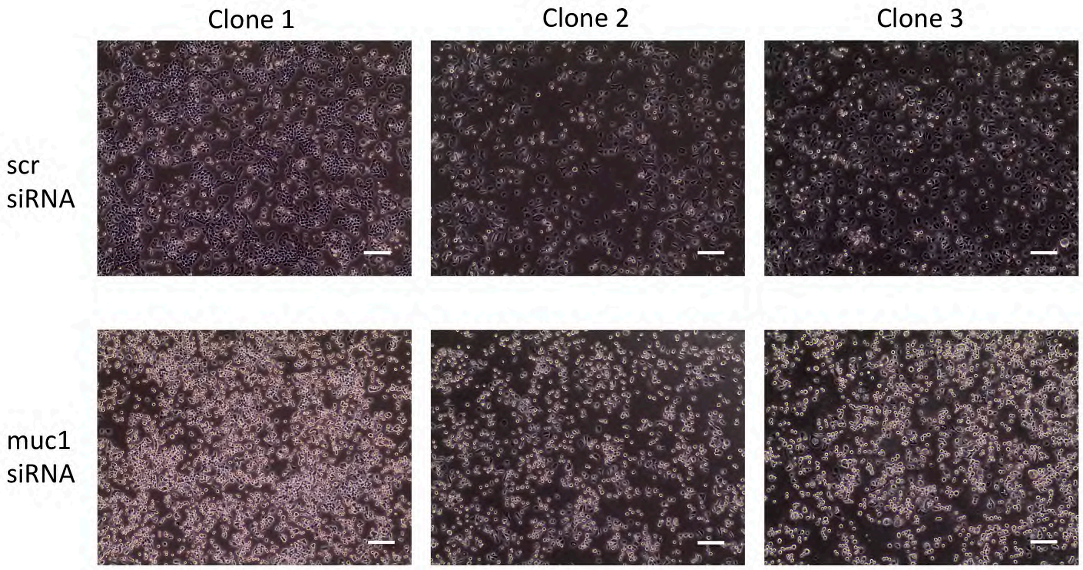

Supplementary Figure 4

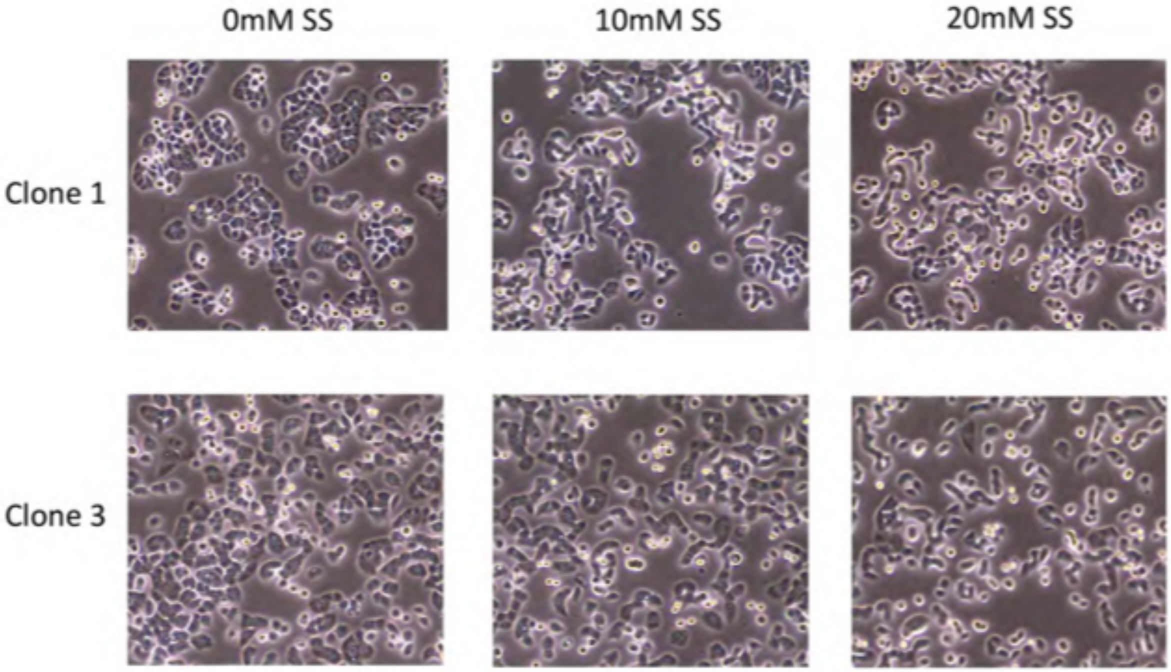

Supplementary Figure 5

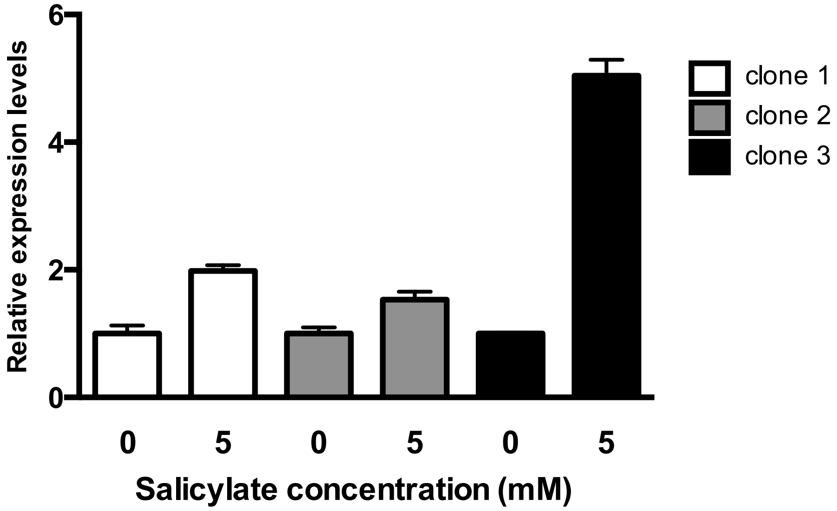

Supplementary Figure 6

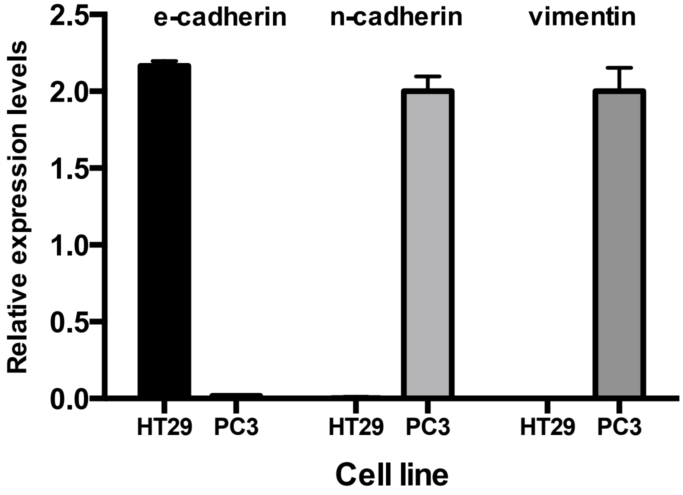

Supplementary Figure 7

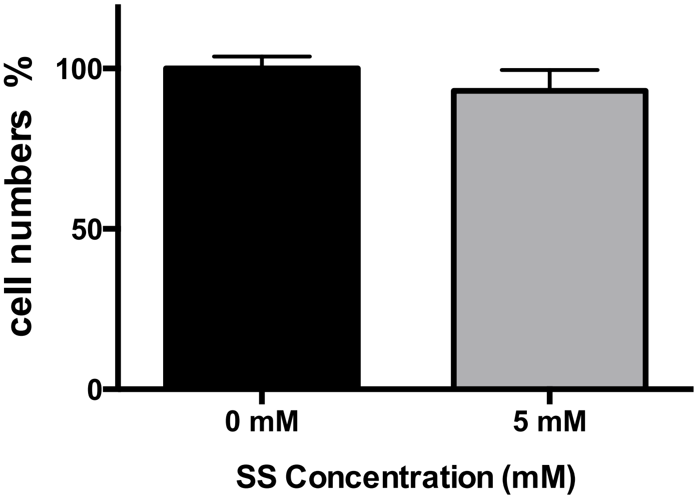

Supplementary Figure 8a

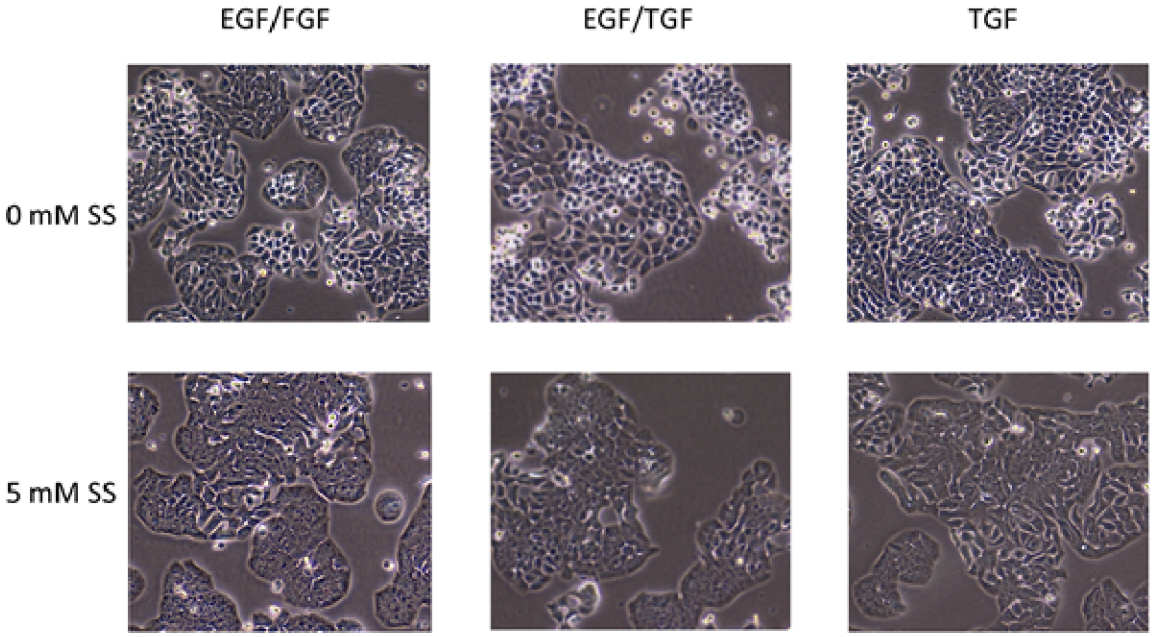

Supplementary Figure 8b

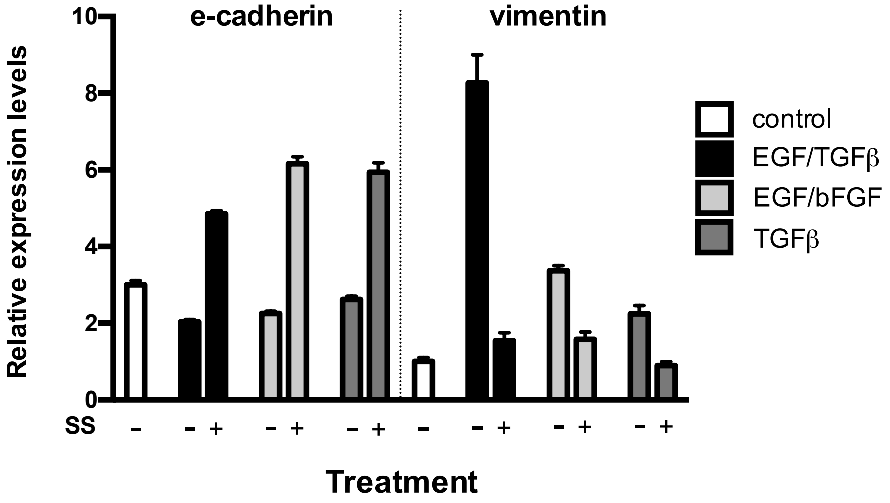

Supplementary Figure 8c

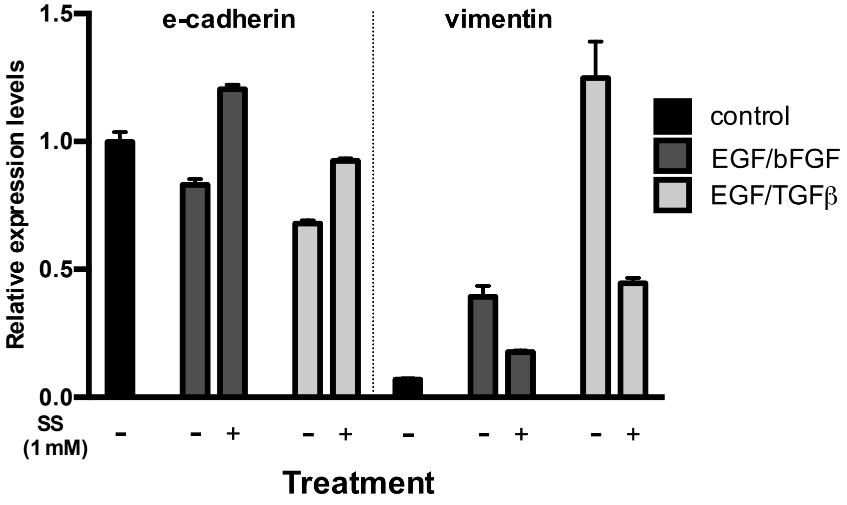

Supplementary Figure 9a

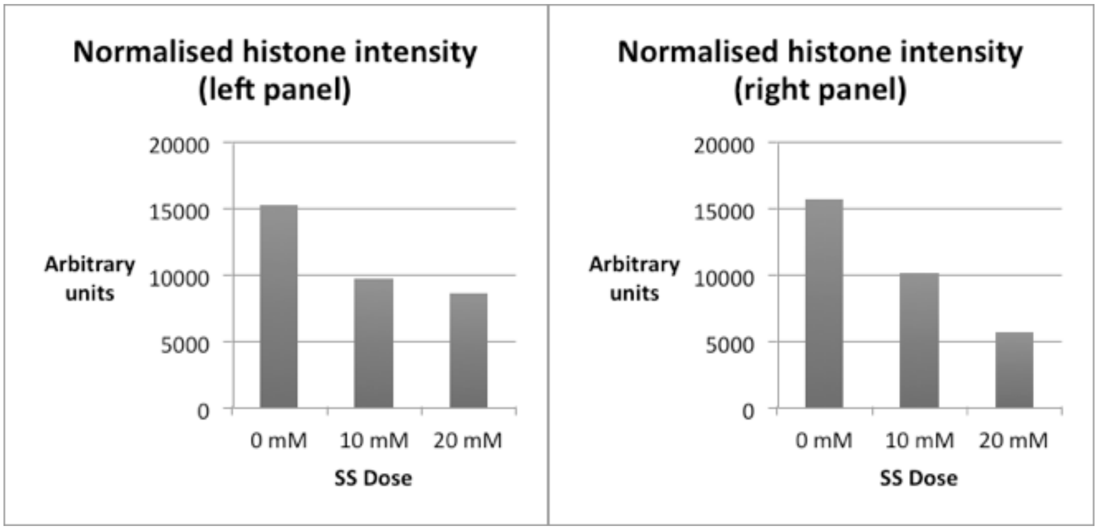

Supplementary Figure 9b

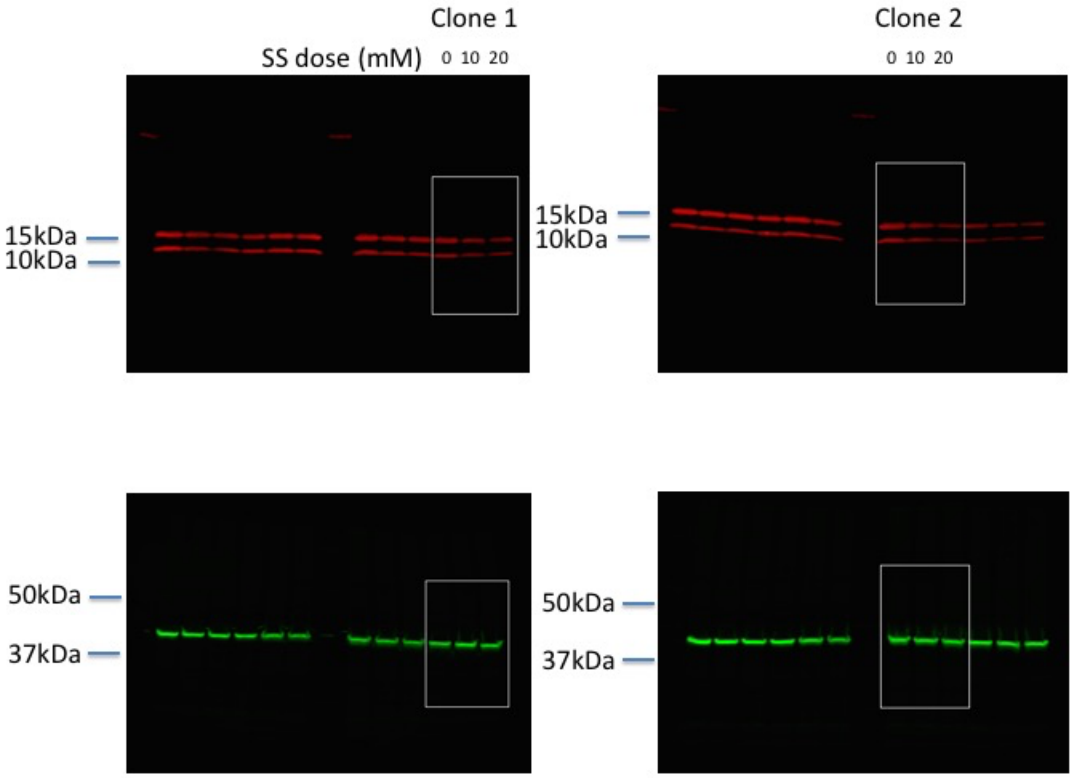

1 Linden, S. K. *et al.* MUC1 limits Helicobacter pylori infection both by steric hindrance and by acting as a releasable decoy. *PLoS pathogens* **5**, e1000617, doi:10.1371/journal.ppat.1000617 (2009).
